# Snapping for 4D‐Printed Insect‐Scale Metal‐Jumper

**DOI:** 10.1002/advs.202307088

**Published:** 2023-11-23

**Authors:** Yang Yang, Yongquan Wang

**Affiliations:** ^1^ School of Mechanical Engineering Xi'an Jiaotong University Xi'an 710049 P. R. China

**Keywords:** 4D printing, bistable structure, jumping, photo‐driven technique, shape memory alloys, snapping mechanism

## Abstract

The replication of jumping motions observed in small organisms poses a significant challenge due to size‐related effects. Shape memory alloys (SMAs) exhibit a superior work‐to‐weight ratio, making them suitable for jumping actuators. However, the SMAs advantages are hindered by the limitations imposed by their single actuator configuration and slow response speed. This study proposes a novel design approach for an insect‐scale shape memory alloy jumper (net‐shell) using 4D printing technology and the bistable power amplification mechanism. The energy variations of the SMA net‐shell under different states and loads are qualitatively elucidated through a spring‐mass model. To optimize the performance of the SMA net‐shell, a non‐contact photo‐driven technique is employed to induce its shape transition. Experimental investigations explore the deformation response, energy release of the net‐shell, and the relationship between the light power density. The results demonstrate that the SMA net‐shell exhibits remarkable jumping capabilities, achieving a jump height of 60 body lengths and takeoff speeds of up to 300 body lengths per second. Furthermore, two illustrative cases highlight the potential of net‐shells for applications in unstructured terrains. This research contributes to miniaturized jumping mechanisms by providing a new design approach integrating smart materials and advanced structures.

## Graphical abstract

This study employs selective laser melting to fabricate an integrated shape memory alloy net‐shell. The net‐shell can mimic the beetle in nature, utilizing its body for power amplification and achieving astonishing jumping performance. The SMA jumper not only mimics natural creatures in function but matches or exceeds them in size, weight, and jumping performance.

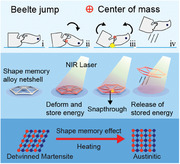

## 1 Introduction

Inspired by nature, soft‐bodied robots exhibit high compliance, enabling them to adapt effectively to diverse environments. Recent advancements in materials and actuation mechanisms have facilitated the emulation of various movements displayed by natural organisms, including flying,^[^ ^1^, ^2^ ^]^ crawling,^[^ ^3^, ^4^, ^5^ ^]^ jumping,^[^ ^6^, ^7^, ^8^, ^9^ ^]^ and swimming.^[^ ^10^, ^11^ ^]^ Among these locomotor modes, jumping has garnered considerable interest as an efficient means of traversing unstructured environments, particularly for small insects seeking survival space. Nevertheless, due to size‐related effects, replicating the jumping motion of diminutive organisms poses significant challenges for soft‐bodied robots.

**Figure 1:**
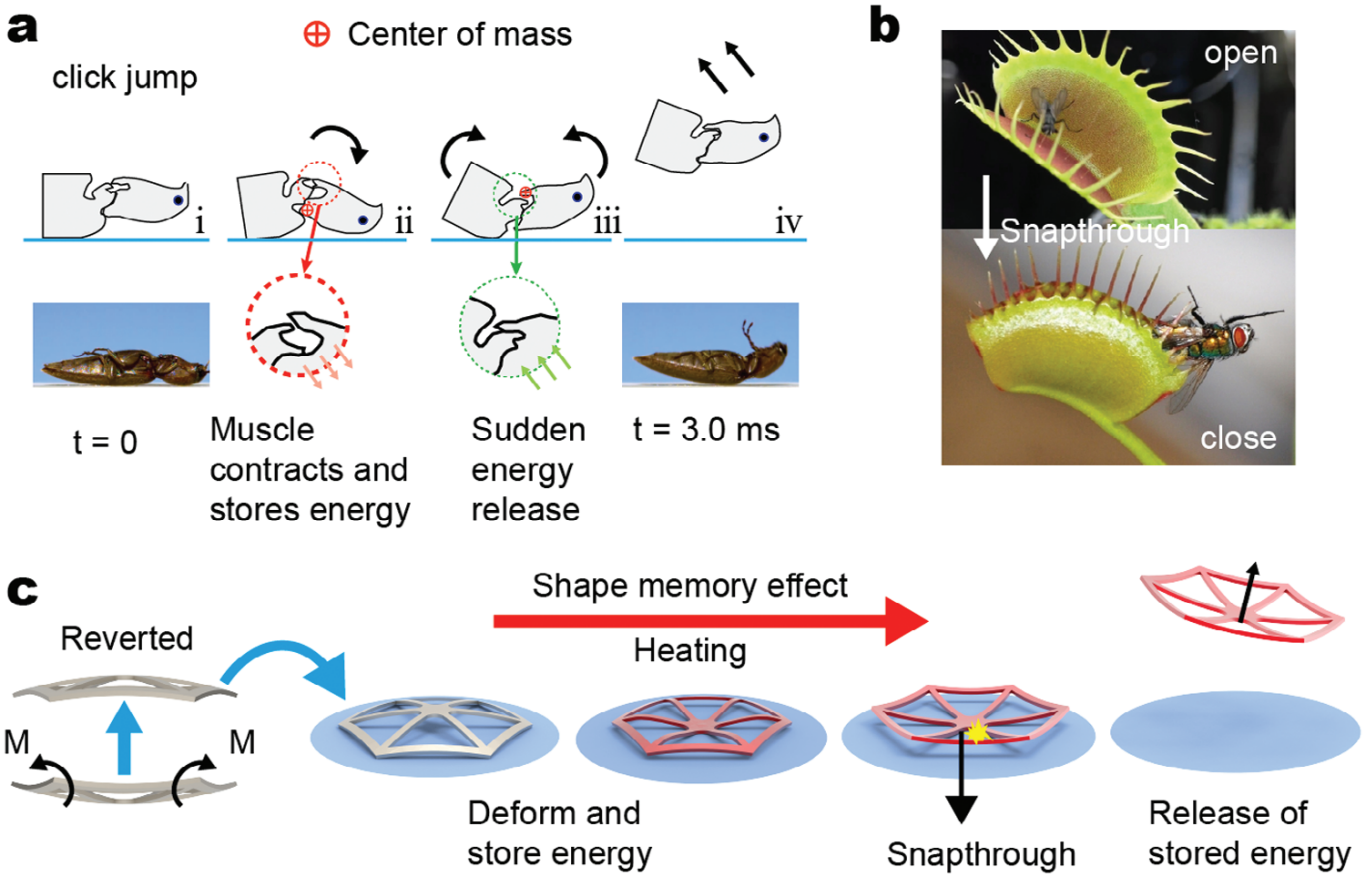
Bionic design and the principle of jump implementation of the SMA net‐shell. a) The click jumping mechanism of the beetle. Reproduced with permission.^[^ ^7^ ^]^ Copyright 2023, The Authors, published by the Proceedings of the National Academy of Sciences of the United States of America. b) Venus flytrap fast closing blades to catch prey. c) Jumping operation of the SMA net‐shell.

In contrast to conventional bulky actuators, lightweight and efficient responsive actuators have the potential to overcome size and mass limitations. Responsive materials can undergo substantial deformations in response to external stimuli, such as light, heat, electricity, or magnetism. Integrating smart actuators with soft robots has yielded a plethora of intelligent small‐scale soft robots exhibiting remarkable performance.^[^ ^12^, ^13^, ^14^, ^15^, ^16^ ^]^ Among these, electrically stimulated soft robots exhibit rapid response speeds, making them suitable candidates for jumping applications.^[^ ^17^, ^18^ ^]^ Nonetheless, direct energy output jumping robots encounter challenges associated with limited instantaneous power output resulting from energy transfer inefficiencies within the system. Consequently, their jumping height tends to be modest. In nature, organisms often employ snapping mechanisms as a means of power amplification. For instance, beetles utilize their bodies to store energy,^[^ ^19^ ^]^ which, once accumulated to a critical level, triggers a snapping transformation, facilitating the rapid release of stored energy and enabling high jumps (**Figure** **1**a). Inspired by such natural phenomena, Wang^[^ ^7^ ^]^ et al. developed an insect‐scale jumping robot that rivals or surpasses natural creatures and existing robotic counterparts. The snapping mechanism obviates the need for high response speeds in the actuators. Consequently, light‐stimulated and thermally stimulated responsive materials, such as liquid crystal elastomers, shape memory polymers, and hydrogels, can be harnessed effectively within jumping robots.^[^ ^20^, ^21^, ^22^, ^23^, ^24^ ^]^

The jump height of a responsive jumping robot is primarily determined by the energy output per unit mass of the intelligent actuator employed. Shape memory alloys (SMAs) exhibit a favorable work‐to‐weight ratio compared to other smart materials,^[^ ^25^ ^]^ rendering them well‐suited for small‐scale jumping robots. SMAs are metallic materials that can spontaneously recover or maintain their memory shape when subjected to heating. However, the response speed of SMAs as thermally responsive materials is inherently limited. Once again, the snapping mechanism can be employed to enhance the instantaneous power output of SMAs. Notably, the configuration of existing SMA actuators often relies on 1D wires and springs^[^ ^26^ ^]^ due to constraints imposed by traditional processing methods. Achieving a snapping mechanism solely with 1D configurations presents challenges, necessitating the incorporation of additional mechanisms such as elastic laminations,^[^ ^16^, ^27^, ^28^ ^]^ flexible hinges,^[^ ^29^, ^30^ ^]^ rigid linkages,^[^ ^6^, ^31^ ^]^ and curved metal laminations.^[^ ^32^ ^]^ These additions increase the system's complexity and hinder the exploitation of the inherent advantages of the significant work‐to‐weight ratio offered by SMAs.

Selective laser melting (SLM) represents an advanced additive manufacturing technique^[^ ^33^ ^]^ wherein the laser energy is employed to selectively melt powdered materials layer‐by‐layer, enabling the fabrication of intricately shaped 3D objects. Over the past decade, a considerable body of research has emerged regarding utilizing selective laser melting for manufacturing SMAs,^[^ ^34^, ^35^, ^36^ ^]^ commonly known as metallic 4D printing technology, due to the incorporation of smart metallic materials in the 3D printing process. However, the existing research primarily focuses on material formation, with limited emphasis on developing functional devices utilizing 4D printing technology specifically tailored for SMAs.

This paper presents a monolithic insect‐scale net‐shell fabricated by 4D printing technology with shape memory alloy (SMA). The structural design of the net‐shell draws inspiration from the Venus flytrap,^[^ ^37^ ^]^ a plant known for its snapping characteristics. The curved blades of the Venus flytrap possess two stable states, namely open and closed, wherein the accumulation of internal energy triggers the rapid closure of the blades to capture prey (Figure 1b). Leveraging this principle, the proposed net‐shell made of shape‐memory alloy exhibits the same structural properties, enabling rapid deformation without needing external auxiliary structures. The net‐shell autonomously reverts to its memory through the shape memory effect, exhibiting a gradual energy accumulation phase followed by a transient release phase, akin to the click jump observed in beetles (Figure 1c). Furthermore, the SMA jumper exhibits an exceptional leaping capacity surpassing that observed in typical insect locomotion.

## 2 Results and Discussion

### 2.1 Operation Principle and Fabrication

**Figure 2:**
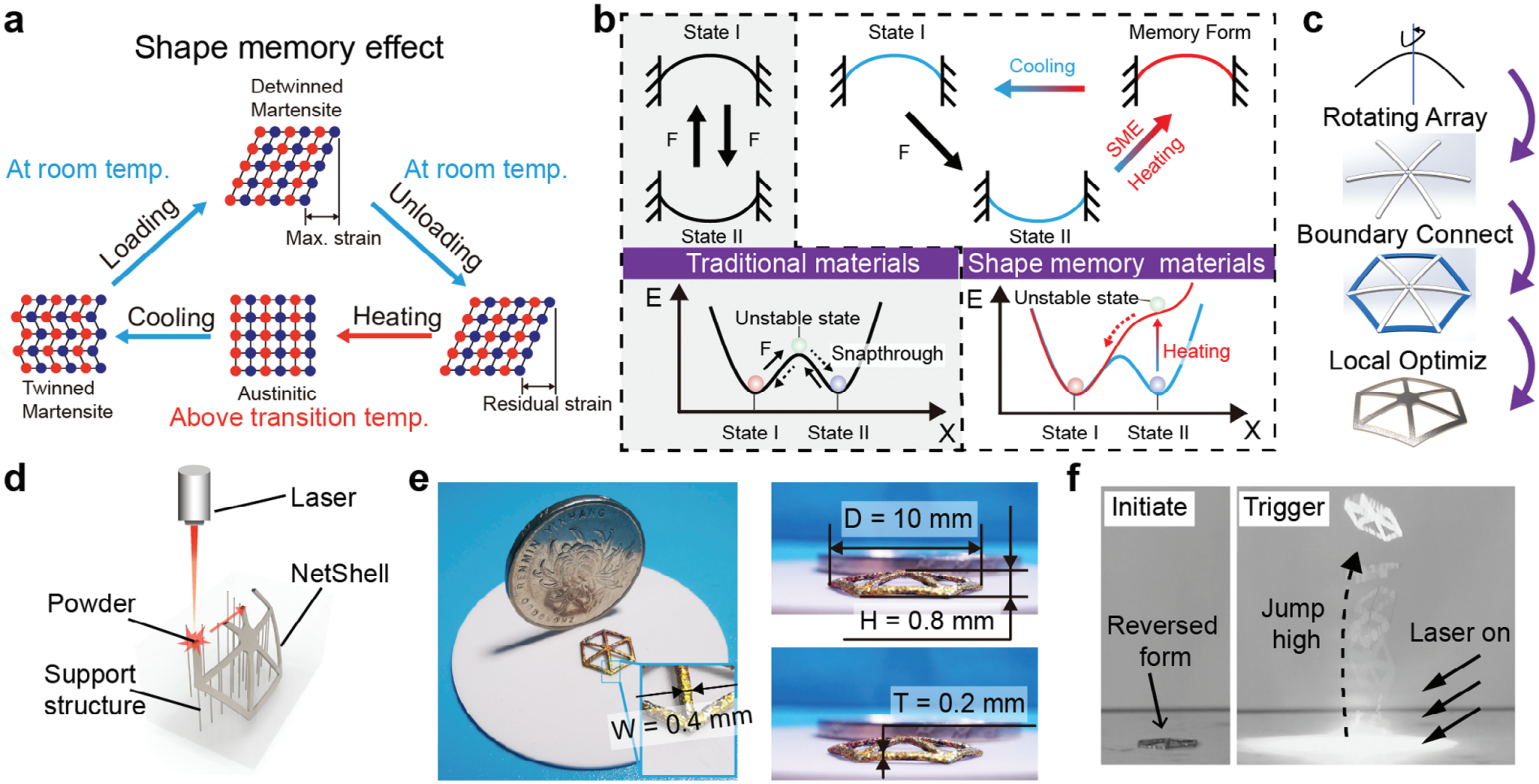
Design and fabrication of shape memory alloy net‐shell structure. a) Microscopic explanation of the shape memory effect. b) Differences in deformation processes of bistable structures made of shape memory materials and traditional materials. c) Structural design steps of the net‐shell. d) Schematic diagram of selective laser melting for manufacturing shape memory alloys. e) Photograph of the shape memory alloy net‐shell. f) Laser drives net‐shell jump.

The shape memory effect can be comprehended through the microstructural modifications occurring within the material (**Figure** **2**a). Under low temperature conditions, the SMA assumes the martensitic phase. Wherein the twin‐crystal martensite transforms into detwined martensite upon loading, then generates residual strain upon unloading. Upon reaching the phase transition temperature, the detwined martensite reverts back to austenite, eliminating the residual strain and the macroscopic restoration of the SMA to its original form. An intelligent bistable system with distinctive functionalities can be achieved by harnessing the dual‐phase states of austenite and detwined martensite in conjunction with bistable configurations.

Conventional materials typically necessitate external forces to facilitate stable‐state bidirectional switching, as their energy curves remain unalterable post‐molding. In contrast, bistable structures constructed using shape‐memory smart materials exhibit energy curves contingent upon environmental stimuli (Figure 2b). For instance, in thermally driven shape‐memory materials, the smart bistability can spontaneously transition to the initial state during the return journey propelled by an external stimulus. This phenomenon stems from the transformation of the energy profile of the smart bistable structure, transitioning from a double potential well to a single potential well upon an increase in temperature. Following the energy minimum theory, the system will naturally descend to the energy minimum state (i.e., the memory form).

The net‐shell structure proposed in this study is derived from the curved beam commonly employed in the bistable system (Figure 2c). The rotation array of curved beams is first applied, and the ends of the rotated curved beams are connected sequentially to form a monolithic net‐shell structure. Subsequently, local optimization of the net‐shell is undertaken to mitigate the risk of stress concentration in the central region. The structural model, then, is fabricated using a laser powder bed fusion. The printing process is depicted in Figure 2d. In terms of powder material selection, NiTi powder was chosen due to the superior mechanical properties and significant strain exhibited by NiTi‐based shape memory alloy. After post‐processing operations, such as heat treatment and de‐supporting, the final net‐shell with the target shape was successfully obtained. We also printed standard samples and performed tensile tests. The mechanical properties of the standard samples are shown in the Figure S3 (Supporting Information).

A photograph of the net‐shell reveals its size, which is smaller than a coin's, and it has a mass of only 40 mg. The specific dimensions are presented in Figure 2e, wherein *W* represents the beam width, *D* represents the span, *H* represents the arch height, and *T* represents the thickness. This paper showcases one particular specimen that exhibits superior fabrication quality and performance. Figure S4,S5 (Supporting Information) provide a detailed discussion of how structural parameters impact both fabrication quality and performance characteristics.

Given the small‐scale configuration of the net‐shell, mitigating environmental interference and optimizing jumping performance is imperative. A contactless optical driving method is employed to achieve this, enabling the net‐shell to be triggered for upward jumping. The printed net‐shell's initial configuration represents the material's memory shape. In the jumping test, it is necessary to revert the form of the net‐shell before placing it on the ground. Under the influence of photothermal effects, the SMA net‐shell undergoes a temperature‐induced phase transition, resulting in shape recovery deformation. During the shape recovery process, the net‐shell triggers the snapping mechanism, exhibiting a motion similar to the click jump. The jumping process of the net‐shell is meticulously recorded using a high‐speed camera (Figure 2f), facilitating accurate analysis and assessment.

### 2.2 Spring‐Mass Model

**Figure 3:**
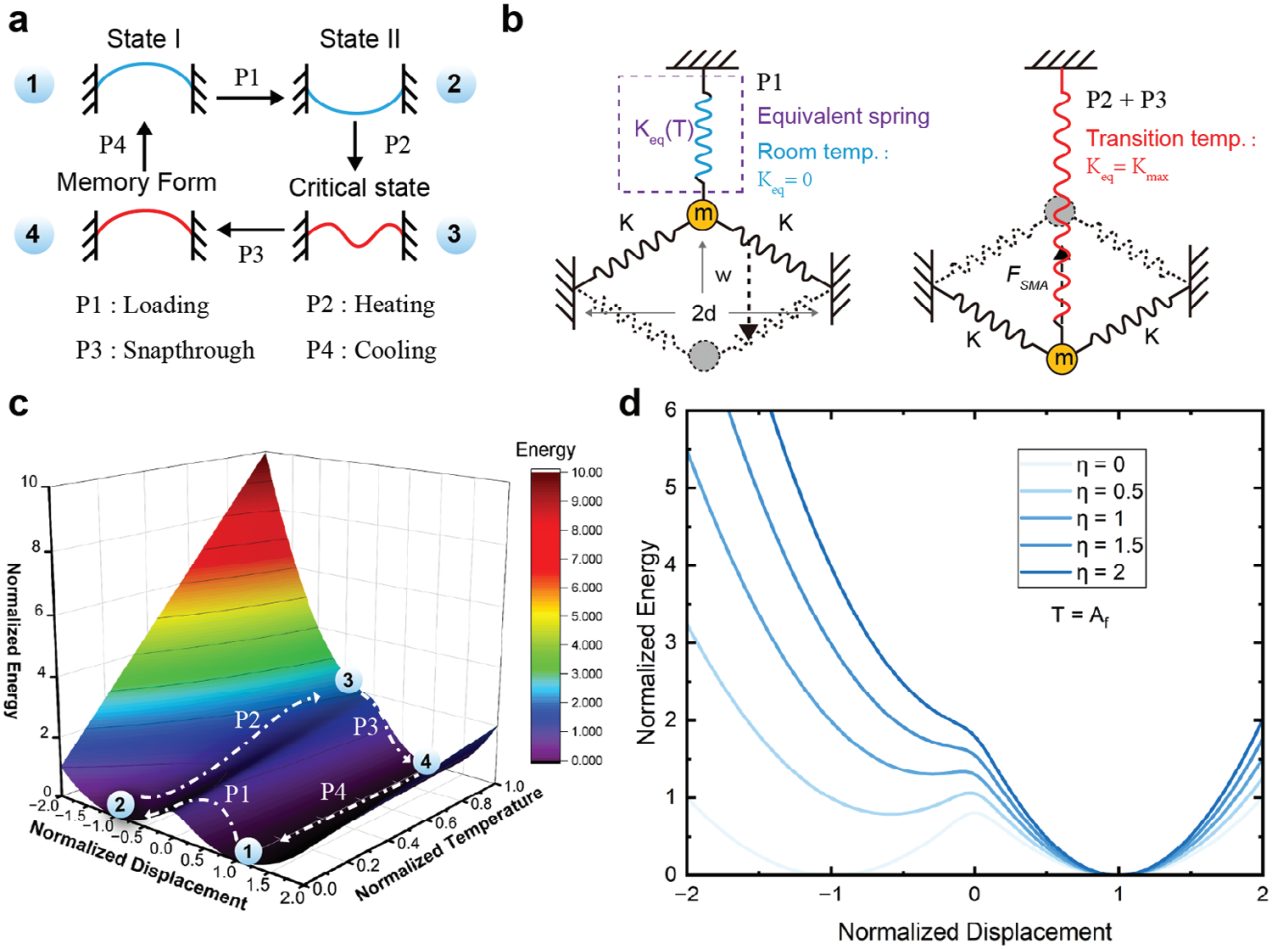
Mathematical model of shape memory bistable system. a) Transformation sequence. b) Spring mass equivalence model. c) 3D energy landscape map. d) Influence of the initial structural stiffness on the energy landscape.

The net‐shell can be simplified as a curved beam structure, as shown in **Figure** **3**a, with a specific deformation and loading sequence. The deformation mechanism of the SMA bistable system can be qualitatively analyzed and explained by employing the spring‐mass model. An equivalent spring with temperature‐dependent stiffness is incorporated to capture the recoverable plastic deformation at low temperatures and the subsequent memory effect following heating exhibited by SMAs (Figure 3b). At lower temperatures, the stiffness of the equivalent spring is zero. As the temperature rises and reaches the austenite start temperature (*A*_s_), the stiffness of the equivalent spring gradually increases. This progressive increment persists until the temperature reaches the austenite finish temperature (*A*_f_), at which point the stiffness of the equivalent spring reaches its maximum value and remains constant after that. The dynamic equation governing this spring‐mass system can be formulated as:

(1) $$m\ddot{w}+2kw\left(1-\frac{L}{\sqrt{w^{2}+d^{2}}}\right)+F_{sma}\left(T\right)=0$$

(2) $$F_{sma}=k_{eq}\left(T\right)\left(\sqrt{L^{2}-d^{2}}-w\right)$$

where *w* represents displacement, *k* represents the initial structural stiffness, *L* represents the original length of the spring, *d* represents the span, and *F*_sma_ represents the restoring force of the shape memory effect. This recovery force depends on temperature and strain, and the relationship is quite complex. It is assumed that the recovery force is linearly related to temperature and strain to simplify the analysis. The equivalent stiffness is expressed through the following equation:

(3) $$k_{eq}\left(T\right)=k_{max}\;\frac{T-A_{s}}{A_{f}-A_{s}}$$

The energy of the whole system can be expressed as:

(4) $$E=k{\left(\sqrt{w^{2}+d^{2}}-L\right)}^{2}+\frac{1}{2}k_{eq}\left(T\right){\left(\sqrt{L^{2}-d^{2}}-w\right)}^{2}$$

Normalizing the energy equation:

(5) $$E_{norm}={\left(\sqrt{w_{norm}^{2}+\alpha^{2}}-1\right)}^{2}+\frac{1}{2}\eta T_{norm}{\left(\sqrt{1^{2}-\alpha^{2}}-w_{norm}\right)}^{2}$$

where $\alpha =\frac{d}{L}$, $\eta =\frac{k_{\max}}{k}$, and $T_{norm}=\frac{T-A_{s}}{A_{f}-A_{s}}$.

A systematic exploration of the normalized temperature parameter generates a 3D energy landscape map for the shape‐memory bistable system (Figure 3c). This comprehensive representation succinctly captures the variations in energy across the four distinct loading paths. With the increase in temperature, the energy landscape undergoes a remarkable transformation from a dual potential well to a singular potential well, thereby elucidating the underlying thermal‐induced snap‐through mechanism inherent in the shape‐memory bistable structure. However, it is important to note that not all cases exhibit thermal‐induced snap‐through behavior. In cases involving a complete phase transition, the energy landscape of the shape memory bistable system can still manifest a dual potential well for small values of *η* (Figure 3d). Consequently, it is imperative to exercise caution in determining the initial structural stiffness, as excessively large values may impede the attainment of a smooth thermal recovery snap‐through. In addition, Figure S6 (Supporting Information) also discusses the impact of shape memory effects on net‐shell performance.

### 2.3 Photothermal Performance

The photothermal effect is a well‐known phenomenon wherein the interaction of electromagnetic radiation with a material leads to heat generation. The photothermal properties of substances vary due to their unique abilities to absorb and reflect light. Substances with higher light energy absorption demonstrate stronger photothermal characteristics and rapid heating. The photothermal performance of a material relies on its absorption capability and conversion efficiency concerning different wavelengths of light. Generally, infrared light is readily absorbed and converted into heat energy within the visible range. However, certain specialized substances, such as specific metals (e.g., NiTi discussed in this paper), may display enhanced photothermal effects when exposed to UV light. Nevertheless, UV light comprises high‐energy electromagnetic waves that can harm the human body. Hence, for reasons of commonality and safety, near‐infrared light (NIR) is chosen as the driving source for shape memory alloys (SMA) in this study.

**Figure 4:**
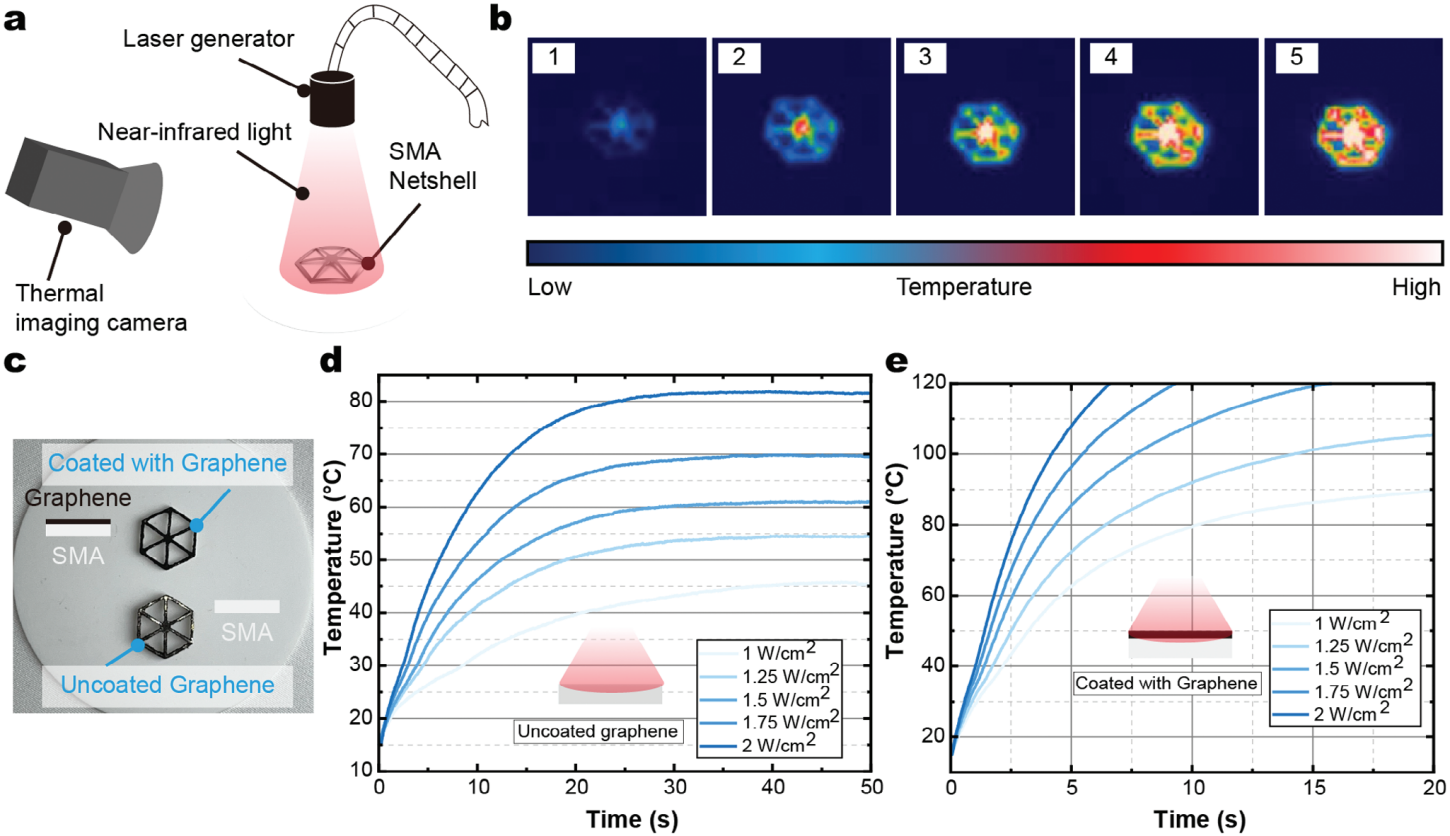
Photothermal testing of the SMA net‐shells. a) Schematic diagram of the testing setup. b) Thermal image of the heating procedure. c) Snapshot of the net‐shells. One is coated with graphene and the other is uncoated with graphene. d,e) Temperature rise curve of the net‐shells under different radiation density.

The experimental setup employed to assess the photothermal performance is depicted in **Figure** **4**a. A laser generator is utilized to induce heating of the SMA net‐shell from the uppermost direction. To facilitate temperature measurement, the SMA net‐shell keeps its memory form, which means that there is no deformation during the heating process. Subsequently, a thermal imaging camera was employed to meticulously record the alterations in surface temperature across the SMA net‐shell (Figure 4b). Upon exposure to infrared laser irradiation, the temperature in the central region of the SMA net‐shell increased first, followed by a subsequent increase in the surrounding temperature. This observable phenomenon is primarily attributed to laser energy's non‐uniform distribution, which conforms to a Gaussian or normal distribution. Accordingly, the central region exhibits the highest energy concentration, while the energy gradually diminishes toward the periphery.

A coating of graphene paste was applied to the surface of the net‐shell to enhance the photothermal properties (Figure 4c). Due to the complex geometry of the net‐shell, obtaining an average temperature is challenging. Therefore, we assessed the photothermal performance of the SMA net‐shell by using the maximum temperature within the detection area (Figure 4d,e). Figure 4d displays the temperature rise curve of the pure SMA net‐shell at various radiation densities. It can be observed that with increasing light density, the warming rate of the pure net‐shell increases, along with a rise in the stabilization temperature. However, even under high light density conditions (2 W cm^−2^), the pure net‐shell requires 30 s to reach a temperature of 80 °C. Because the laser can only heat the surface of SMA, and then considering the heat transfer loss in the thickness direction, the surface reference temperature is greater than the transition temperature (60 °C). Conversely, the net‐shell coated with graphene exhibits a significantly higher heating rate (Figure 4e). With the same light power density (2 W cm^−2^), the surface‐modified net‐shell only necessitates ≈3 s to attain a temperature of 80 °C. Even at a lower power density (1 W cm^−2^), the ramp‐up time to 80 °C is a mere 10 s. This notable improvement is primarily attributed to the outstanding photothermal performance of graphene.^[^ ^38^ ^]^ Consequently, the subsequent experiments exclusively employ the net‐shell with the graphene coating.

### 2.4 Morphing Response and Impact Characteristics

To observe the process of heating‐induced deformation of the net‐shell, the net‐shell, after being inverted, was affixed to a vertically oriented copper column. A laser beam was directed from above to illuminate the net‐shell. The graphene coating applied to the net‐shell swiftly generates substantial heat due to the photothermal effect. Of graphene's remarkable thermal conductivity, the heat is efficiently transferred to the SMA net‐shell. Consequently, the temperature of the SMA increases, triggering deformation that ultimately leads to restoring its memory shape. Since lasers can obscure objects in grayscale camera shots, a high‐speed color camera was chosen to capture and document the heating‐induced deformation process.

**Figure 5:**
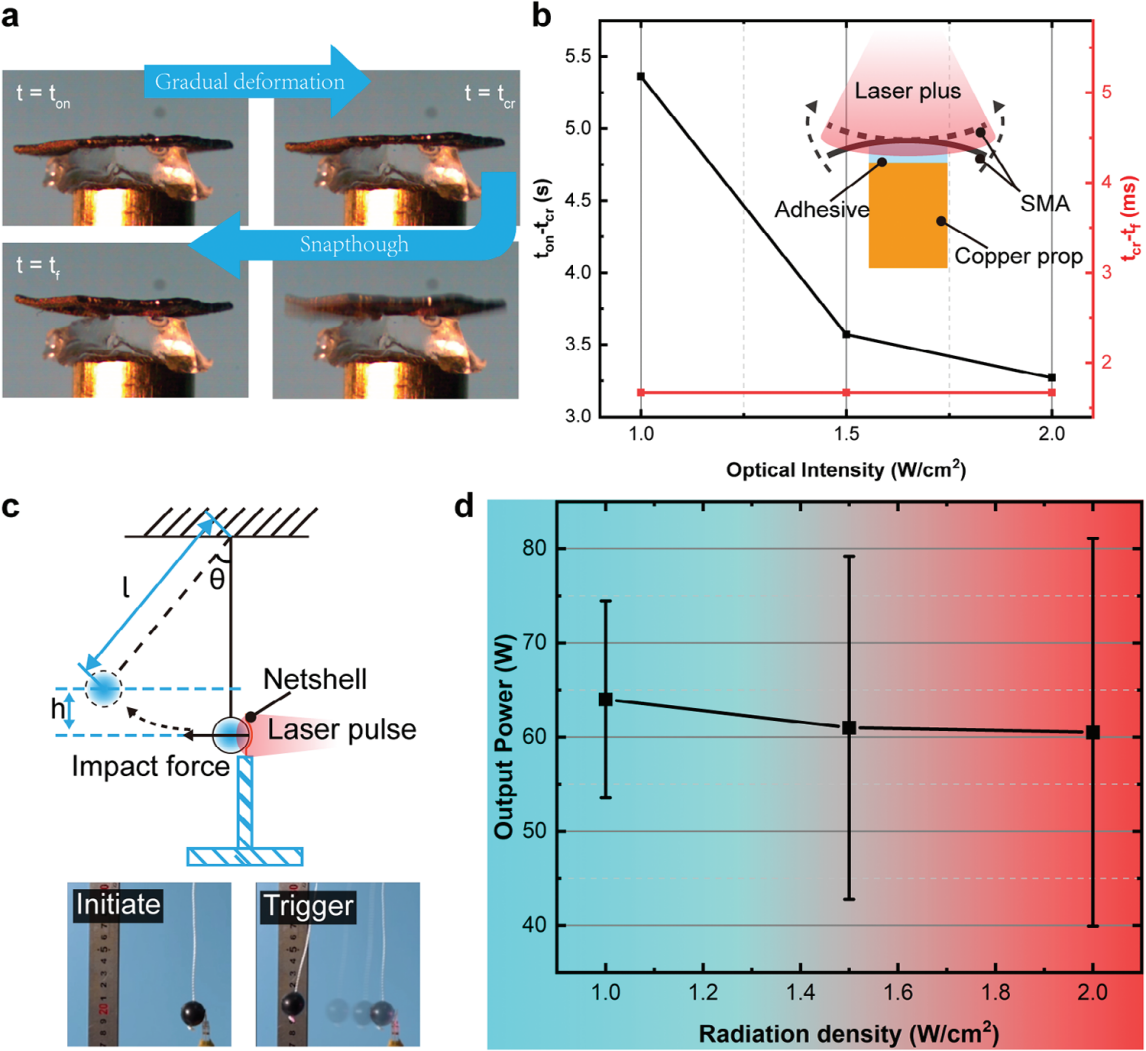
Morphing response and impact characteristics. a) Deformation process. b) Deformation response time under different radiation densities. c) Pendulum experiment schematic and test graph. (d) Impact power at different radiation densities.

The complete deformation process of the net‐shell (Movie S1, Supporting Information) was effectively captured by a high‐speed camera operating at a frame rate of 3000 frames per second (FPS). The process exhibited two distinct stages, as illustrated in **Figure** **5**a. The first stage involved gradual and minor deformation, while the subsequent stage exhibited rapid and significant deformation, known as snap‐through. The duration of the first stage deformation is referred to as the preparation time, while the duration of the second stage deformation is denoted as the snapping time. Experimental findings reveal a notable reduction in the preparation time with increasing irradiation density, while the snapping time remains consistently below 2 ms for various irradiation densities (Figure 5b). This behavior is attributed to the fact that the first deformation stage represents the energy accumulation process within the net‐shell. The energy barrier required for accumulation remains unchanged under identical structural and loading conditions. Consequently, the increase in irradiation density facilitates a higher rate of energy accumulation, resulting in a decreased preparation time. On the other hand, the snapping deformation represents the energy release process, primarily influenced by the net‐shell's structural configuration. However, due to the limitations imposed by the current frame rate, the precise relationship between the snapping time and irradiation density cannot be accurately determined. Nevertheless, the experimental results conclusively demonstrate that the snapping time remains remarkably short under different irradiation densities, thus affirming the viability of harnessing structural instability to enhance the response speed of shape memory alloys (SMAs).

The rapid deformation process of the net‐shell is accompanied by the storage and subsequent release of energy. A pendulum experiment was conducted to analyze the instantaneous energy release of the net‐shell quantitatively. The experimental setup is depicted schematically in Figure 5c. The inverted net‐shell, subjected to laser irradiation, undergoes rapid deformation and strikes a small ball, causing it to exhibit a certain degree of oscillatory motion (Movie S2, Supporting Information). By applying the principle of energy conservation, it becomes possible to approximate the energy released instantaneously of the net‐shell. We used the snapping time as the impact duration and the increment in gravitational potential energy of the pendulum mass as the impact energy to calculate the impact power. As depicted in Figure 5, the average impact power of the net‐shell can reach a substantial 60 W, which highlights the advantages of combining NiTi material with a bistable structural configuration. Furthermore, the calculated results demonstrate that the output power of the net‐shell remains unaffected by the irradiation density.

### 2.5 Jumping Capability

**Figure 6:**
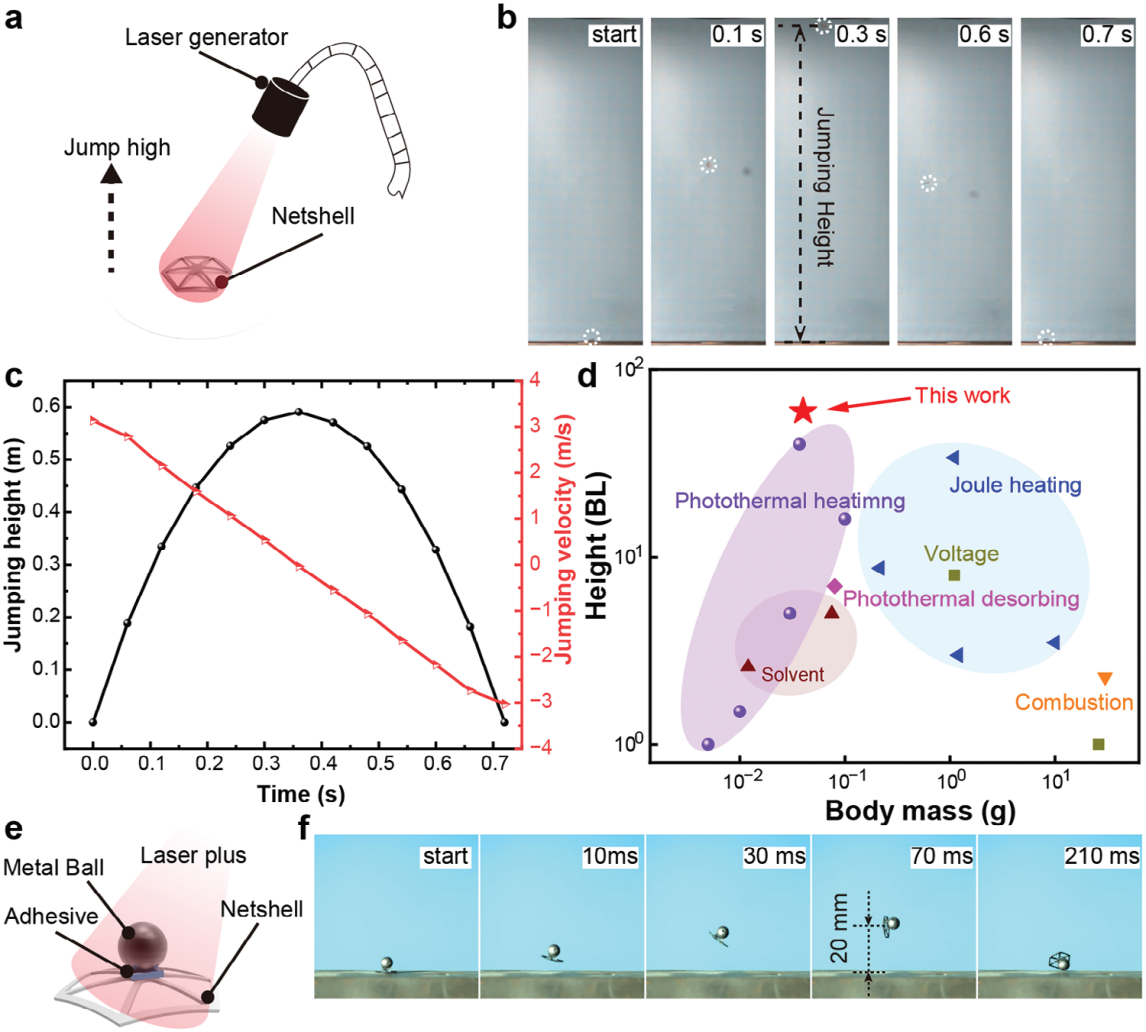
Jumping tests of the net‐shells. a) Schematic diagram of the setup for free jumping. b) Free jumping process. c) Free jumping performance. d) Jumping height comparison with the reported miniature intelligent jumping robots categorized by actuation methods (voltage,^[^ ^8^, ^18^ ^]^ photothermal heating,^[^ ^9^, ^15^, ^20^, ^22^, ^23^ ^]^ solvent,^[^ ^39^, ^40^ ^]^ combustion,^[^ ^41^ ^]^ photothermal desorbing,^[^ ^42^ ^]^ and joule heating^[^ ^27^, ^29^, ^43^ ^]^). e) Schematic diagram of the setup for load jumping. f) The process of load jumping.

The SMA net‐shell possesses distinctive characteristics, including rapid deformation and instantaneous energy release, rendering it highly suitable for applications in the field of jumping. Free jumping tests were conducted using the net‐shell to assess its jumping capabilities, and the experimental setup is depicted in **Figure** **6**a. Placing the inverted net‐shell on the ground, it underwent rapid shape transition upon light stimulation. During this transformative process, the net‐shell forcefully impacted the ground, resulting in an upward leap propelled by the reactive force exerted on the net‐shell (Movie S3, Supporting Information). The entire jumping process of the net‐shell, encompassing takeoff, ascent, descent, and landing, was meticulously captured by a high‐speed camera, as depicted in Figure 6b. Calculations results determined that the net‐shell achieved a remarkable takeoff velocity of 3 m s^−1^, accompanied by an impressive jumping height of up to 600 mm (Figure 6c), which is 60 times its body length scale. This remarkable jumping performance surpasses that of natural organisms. Comparative to other intelligent biomimetic jumpers, this work demonstrates the dual advantages of lightweight construction and high jumping capabilities (Figure 6d). Thus, it can be asserted that the net‐shell effectively showcases the material advantages inherent in SMA.

Nevertheless, the jumping performance of the net‐shell experiences a slight decline under load conditions (Movie S4, Support Information). Specifically, a metallic ball with a mass of 0.4 g, which is ten times heavier than the net‐shell itself, was firmly affixed to its center using adhesive (Figure 6e). Subsequently, when subjected to light stimulation, the net‐shell exhibited a modest leap of ≈20 mm (Figure 6f). This reduction in jumping height can be ascribed to two factors. First, introducing the additional load increases overall weight, impeding the net‐shell's ability to achieve higher jumps. Second, it is conceivable that the presence of the metallic ball obstructed the passage of light in the central region of the net‐shell, leading to an uneven distribution of heat across its surface. Consequently, this uneven heating pattern negatively impacts the net‐shell's recovery path and diminishes its overall bouncing performance.

### 2.6 Unstructured Environment Testing

The jumping motion exhibits efficient locomotion capabilities in unstructured terrains, including underwater and sandy environments. This research paper substantiates the potential application of the net‐shell's stimulus‐responsive jumping through two comprehensive case studies.

**Figure 7:**
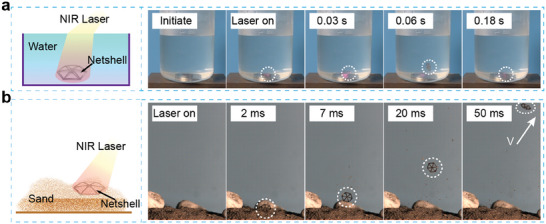
Two case studies. a) Bouncing underwater. b) Leaping out of the sand surface.

First, an underwater scenario test was conducted to validate its viability (Movie S5, Support Information). The inverted net‐shell was positioned in water, and laser was employed to illuminate the surface of the SMA net‐shell after penetrating the water medium. The optical power was appropriately augmented during the experiment to compensate for laser energy absorption by water, as lower optical power failed to elicit the desired jumping response from the net‐shell. Due to the increased underwater resistance, the net‐shell achieved a modest leap of 10 mm (**Figure** **7**a). Nonetheless, this case study exemplified the potential utilization of SMA bistable structures for remote actuation in underwater settings. The development of innovative and noiseless underwater propellers becomes a feasible prospect through meticulous design modifications aimed at enhancing surface tension.

Subsequently, a sandy terrain scenario test was conducted to evaluate the net‐shell's performance further (Movie S6, Supporting Information). The inverted net‐shell was carefully buried beneath the sandy surface, and laser irradiation on the sand surface facilitated an elevation in sand temperature. Subsequent thermal conduction to the net‐shell induced a temperature‐driven phase transition, ultimately instigating an upward leap from beneath the sandy substrate (Figure 7b). Despite the loose and irregular nature of the sandy ground, the net‐shell exhibited a takeoff velocity equivalent to that observed on a flat surface, reaching 2.9 m s^−1^. This case study convincingly illustrated the net‐shell's remarkable adaptability to challenging environmental conditions.

## 3 Conclusion

This study introduces a novel design strategy that combines shape memory alloys (SMA) with bistable structures using metal additive manufacturing technology. By doing so, the limitations associated with size‐related effects and slow material response are effectively overcome, resulting in the development of a millimeter‐scale SMA jumper. The feasibility of this design concept was substantiated through mathematical modeling and experimental validation. In comparison to existing SMA‐driven actuators, the SMA jumper stands out by achieving the integration of structure and functionality, breaking away from the confines of conventional single actuation forms. In order to optimize its jumping capability, a non‐contact optical actuation method was employed. The SMA jumper demonstrates exceptional jumping performance and robust adaptability to varying environmental conditions. This research contributes to advancing design strategies for miniaturized jumping devices, showcasing the potential of SMA technology in creating agile and versatile mechanisms for diverse applications.

However, the limitations of current printing precision hinder the fabrication of smaller‐scale and more intricate SMA structures. By leveraging higher‐precision printing techniques,^[^ ^44^ ^]^ the development of micrometer‐level functional devices emerges as a highly promising trajectory for future exploration. This work will serve as a catalyst for the fusion of smart materials and advanced structural designs, thereby opening up avenues for the creation of innovative and extraordinary functional devices.

## 4 Experimental Section

### Fabrication the SMA Net‐shell

The EP‐M300 machine was equipped with a maximum 1000 W fiber laser beam with a 50 µm diameter. For the LPBF process, process parameters were opted that included a laser power of 200 W, a scanning speed of 1000 mm s^−1^, a powder bed layer thickness of 50 µm, and a hatch spacing of 60 µm. To lay down the layers, a scanning pattern called “stripe rotation” (Figure S1, Supporting Information) was used. Commercialized nickel–titanium alloy powder (Asia New Materials (Beijing) Co., Ltd., China) with a nearly equal atomic ratio with particles of 15–53 µm to build NiTi parts on a NiTi substrate maintained at 180 °C was utilized. Once the printing process was completed, the part needs to be annealed for heat treatment to reduce the surface stress. The heat treatment was performed by heating up to 500 °C at a rate of 10 °C min^−1^ and holding time of 0.5 h at this temperature, and finally cooling to room temperature with the furnace.

### Graphene Coating

Commercialized graphene conductive ink (LN‐GCI‐I, Leader Nano, China) was applied to the surface of NiTi SMA. The ink had a graphene content of 30% and had good adhesion properties. Then the graphene‐coated sample was put into a thermostat and held at 40 °C for 4 h, and the graphene ink was completely cured.

### Measurement Equipment

The transition temperature was measured by a differential scanning calorimetry (DSC). The DSC data (Figure S2, Supporting Information) were achieved, and analyzed, by using a Discovery 250 (TA Instruments) with a heating curve performance from −80 to 100 °C and a cooling curve performance from 100 to −80 °C. Both temperature change rates were used with 10 °C min^−1^. The temperature of the SMA was measured by using an infrared camera (FLIR E40). The sampling frequency of the thermal imaging camera was set to 30 FPS, and the accompanying software (FLIR TOOL+) could directly connect to the camera to measure and record data online, and in real‐time. A laser generator (LSR808H‐8W‐FC) equipped with a power supply (LSR‐PS‐FA) and a fiber (S‐400um‐S‐1 m) was used. The laser had a maximum power of 8 W and produces 808 nm light. The monochrome high‐sensitivity high‐speed camera (NAC MemrecamHX‐6) is used to capture the response of the SMA jumper triggered by laser irradiation. The color high‐speed camera (X213) was used to capture the complete deformation response and jumping process of the SMA. The shooting process was captured at 3000 fps.

## Conflict of Interest

The authors declare no conflict of interest.

## Supporting information

Supporting Information

Supporting Movie 1

Supporting Movie 2

Supporting Movie 3

Supporting Movie 4

Supporting Movie 5

Supporting Movie 6

## Data Availability

The data that support the findings of this study are available from the corresponding author upon reasonable request.

## References

[1] T. Helps , C. Romero , M. Taghavi , A. T. Conn , J. Rossiter , Sci Robot 2022, 7, eabi8189. 35108024 10.1126/scirobotics.abi8189

[2] Y. Chen , H. Zhao , J. Mao , P. Chirarattananon , E. F. Helbling , N.‐S. P. Hyun , D. R. Clarke , R. J. Wood , Nature 2019, 575, 324. 31686057 10.1038/s41586-019-1737-7

[3] T. Yao , Y. Wang , B. Zhu , D. Wei , Y. Yang , X. Han , Smart Mater. Struct. 2020, 30, 015018.

[4] S. Wu , Y. Hong , Y. Zhao , J. Yin , Y. Zhu , Sci. Adv. 2023, 9, eadf8014. 36947625 10.1126/sciadv.adf8014 PMC10032605

[5] S. Wu , G. L. Baker , J. Yin , Y. Zhu , Soft Robot. 2022, 9, 1031. 34874763 10.1089/soro.2021.0080

[6] J.‐S. Koh , E. Yang , G.‐P. Jung , S.‐P. Jung , J. H. Son , S.‐I. Lee , P. G. Jablonski , R. J. Wood , H.‐Y. Kim , K.‐J. Cho , Science 2015, 349, 517. 26228144 10.1126/science.aab1637

[7] Y. Wang , Q. Wang , M. Liu , Y. Qin , L. Cheng , O. Bolmin , M. Alleyne , A. Wissa , R. H. Baughman , D. Vella , S. Tawfick , Proc. Natl. Acad. Sci. USA 2023, 120, e2210651120. 36689664 10.1073/pnas.2210651120 PMC9945960

[8] R. Chen , Z. Yuan , J. Guo , L. Bai , X. Zhu , F. Liu , H. Pu , L. Xin , Y. Peng , J. Luo , L. Wen , Y. Sun , Nat. Commun. 2021, 12, 7028. 34876570 10.1038/s41467-021-27265-w PMC8651723

[9] L. Xu , F. Xue , H. Zheng , Q. Ji , C. Qiu , Z. Chen , X. Zhao , P. Li , Y. Hu , Q. Peng , X. He , Nano Energy 2022, 103, 107848.

[10] Y. Chi , Y. Hong , Y. Zhao , Y. Li , J. Yin , Sci. Adv. 2022, 8, eadd3788. 36399554 10.1126/sciadv.add3788 PMC9674291

[11] G. Li , X. Chen , F. Zhou , Y. Liang , Y. Xiao , X. Cao , Z. Zhang , M. Zhang , B. Wu , S. Yin , Y. Xu , H. Fan , Z. Chen , W. Song , W. Yang , B. Pan , J. Hou , W. Zou , S. He , X. Yang , G. Mao , Z. Jia , H. Zhou , T. Li , S. Qu , Z. Xu , Z. Huang , Y. Luo , T. Xie , J. Gu , et al., Nature 2021, 591, 66. 33658693 10.1038/s41586-020-03153-z

[12] C. Mc Caffrey , T. Umedachi , W. Jiang , T. Sasatani , Y. Narusue , R. Niiyama , Y. Kawahara , Soft Rob. 2020, 7, 700. 10.1089/soro.2019.0090 32223590

[13] T. Li , Z. Zou , G. Mao , X. Yang , Y. Liang , C. Li , S. Qu , Z. Suo , W. Yang , Soft Rob. 2019, 6, 133. 10.1089/soro.2018.0053 30407127

[14] M. Han , X. Guo , X. Chen , C. Liang , H. Zhao , Q. Zhang , W. Bai , F. Zhang , H. Wei , C. Wu , Q. Cui , S. Yao , B. Sun , Y. Yang , Q. Yang , Y. Ma , Z. Xue , J. W. Kwak , T. Jin , Q. Tu , E. Song , Z. Tian , Y. Mei , D. Fang , H. Zhang , Y. Huang , Y. Zhang , J. A. Rogers , Sci Robot 2022, 7, eabn0602. 35613299 10.1126/scirobotics.abn0602

[15] J. Jeon , J.‐C. Choi , H. Lee , W. Cho , K. Lee , J. G. Kim , J.‐W. Lee , K.‐I. Joo , M. Cho , H.‐R. Kim , J. J. Wie , Mater. Today 2021, 49, 97.

[16] D. K. Patel , X. Huang , Y. Luo , M. Mungekar , M. K. Jawed , L. Yao , C. Majidi , Adv. Mater. Technol. 2023, 8, 2201259.

[17] E. Acome , S. K. Mitchell , T. G. Morrissey , M. B. Emmett , C. Benjamin , M. King , M. Radakovitz , C. Keplinger , Science 2018, 359, 61. 29302008 10.1126/science.aao6139

[18] S. K. Mitchell , X. Wang , E. Acome , T. Martin , K. Ly , N. Kellaris , V. G. Venkata , C. Keplinger , Adv. Sci. 2019, 6, 1900178. 10.1002/advs.201900178 PMC6662077 31380206

[19] O. Bolmin , J. J. Socha , M. Alleyne , A. C. Dunn , K. Fezzaa , A. A. Wissa , Proc. Natl. Acad. Sci. USA 2021, 118, e2014569118. 33468629 10.1073/pnas.2014569118 PMC7865152

[20] J. Wang , T. Zhao , Y. Fan , H. Wu , J.‐A. Lv , Adv. Funct. Mater. 2023, 33, 2209798.

[21] J. Hu , Z. Nie , M. Wang , Z. Liu , S. Huang , H. Yang , Angew. Chem., Int. Ed. 2023, 62, e202218227. 10.1002/anie.202218227 36624053

[22] H. Guo , A. Priimagi , H. Zeng , Adv. Funct. Mater. 2022, 32, 2108919.

[23] C. Ahn , X. Liang , S. Cai , Adv. Mater. Technol. 2019, 4, 1900185.

[24] G. Gao , Z. Wang , D. Xu , L. Wang , T. Xu , H. Zhang , J. Chen , J. Fu , ACS Appl. Mater. Interfaces 2018, 10, 41724. 30387979 10.1021/acsami.8b16402

[25] J. Mohd Jani , M. Leary , A. Subic , M. A. Gibson , Mater. Des. 2014, 56, 1078.

[26] H. Rodrigue , W. Wang , M.‐W. Han , T. J. Y. Kim , S.‐H. Ahn , Soft Rob. 2017, 4, 3. 10.1089/soro.2016.0008 29182099

[27] S. Nishikawa , Y. Arai , R. Niiyama , Y. Kuniyoshi , IEEE Robot Autom Lett 2018, 3, 1018.

[28] S.‐P. Jung , G.‐P. Jung , J.‐S. Koh , D.‐Y. Lee , K.‐J. Cho , J Mech Robot 2015, 7, 021010.

[29] Z. Zhakypov , K. Mori , K. Hosoda , J. Paik , Nature 2019, 571, 381. 31292552 10.1038/s41586-019-1388-8

[30] R. Kurniawan , T. Fukudome , H. Qiu , M. Takamiya , Y. Kawahara , J. Yang , R. Niiyama , in 2020 IEEE/RSJ Int. Conf. on Intelligent Robots and Systems (IROS), IEEE, Piscataway, NJ 2020, pp. 7881.

[31] D. Li , D. Niu , G. Ye , B. Lei , J. Han , W. Jiang , F. Luo , J. Chen , H. Liu , B. Lu , Appl. Mater. Today 2021, 24, 101091.

[32] Y. Wan , K. Cuff , M. J. Serpe , Adv. Intell. Syst. 2022, 4, 2100251.

[33] T. Debroy , H. L. Wei , J. S. Zuback , T. Mukherjee , J. W. Elmer , J. O. Milewski , A. M. Beese , A. Wilson‐Heid , A. De , W. Zhang , Prog. Mater. Sci. 2018, 92, 112.

[34] M. Elahinia , N. Shayesteh Moghaddam , M. Taheri Andani , A. Amerinatanzi , B. A. Bimber , R. F. Hamilton , Prog. Mater. Sci. 2016, 83, 630.

[35] Z. Xiong , Z. Li , Z. Sun , S. Hao , Y. Yang , M. Li , C. Song , P. Qiu , L. Cui , J. Mater. Sci. Technol. 2019, 35, 2238.

[36] L. Xue , K. C. Atli , C. Zhang , N. Hite , A. Srivastava , A. C. Leff , A. A. Wilson , D. J. Sharar , A. Elwany , R. Arroyave , I. Karaman , Acta Mater. 2022, 229, 117781.

[37] R. Sachse , A. Westermeier , M. Mylo , J. Nadasdi , M. Bischoff , T. Speck , S. Poppinga , Proc. Natl. Acad. Sci. USA 2020, 117, 16035. 32571929 10.1073/pnas.2002707117 PMC7355038

[38] B. Han , Y.‐L. Zhang , Q.‐D. Chen , H.‐B. Sun , Adv. Funct. Mater. 2018, 28, 1802235.

[39] K. Yu , X. Ji , T. Yuan , Y. Cheng , J. Li , X. Hu , Z. Liu , X. Zhou , L. Fang , Adv. Mater. 2021, 33, 2104558. 10.1002/adma.202104558 34514641

[40] H. Lee , C. Xia , N. X. Fang , Soft Matter 2010, 6, 4342.

[41] R. F. Shepherd , A. A. Stokes , J. Freake , J. Barber , P. W. Snyder , A. D. Mazzeo , L. Cademartiri , S. A. Morin , G. M. Whitesides , Angew. Chem., Int. Ed. 2013, 52, 2892. 10.1002/anie.201209540 23382085

[42] Y. Kim , J. Van Den Berg , A. J. Crosby , Nat. Mater. 2021, 20, 1695. 33526877 10.1038/s41563-020-00909-w

[43] M. Noh , S.‐W. Kim , S. An , J.‐S. Koh , K.‐J. Cho , IEEE Trans. Robot. 2012, 28, 1007.

[44] J. Fu , Z. Hu , X. Song , W. Zhai , Y. Long , H. Li , M. Fu , Opt. Laser Technol. 2020, 131, 106374.

