# Supplementary material for: Snapping for 4D‐Printed Insect‐Scale Metal‐Jumper
Source: Adv Sci (Weinh). 2023 Nov 23;11(3):2307088. doi: 10.1002/advs.202307088 (PMC10797476; doi:10.1002/advs.202307088)
Supplement: Supplementary file 1 — Supporting Information [file ADVS-11-2307088-s005.pdf]

ADVANCED SCIENCE
Open Access

Supporting Information

for *Adv. Sci.*, DOI 10.1002/advs.202307088

Snapping for 4D-Printed Insect-Scale Metal-Jumper

*Yang Yang and Yongquan Wang**

Supporting Information

Snapping for 4D printed insect-scale metal-jumper

*Yang Yang, and Yongquan Wang**

Y. Yang, Y. Wang

School of Mechanical Engineering

Xi'an Jiaotong University

Xi'an 710049, People's Repulic of China

E-mail: yqwang@mail.xjtu.edu.cn

The file includes:
Figures S1 to S6

Other supporting information for this manuscript includes:
Movies S1 to S6

1. Scanning strategy for laser powder bed melting

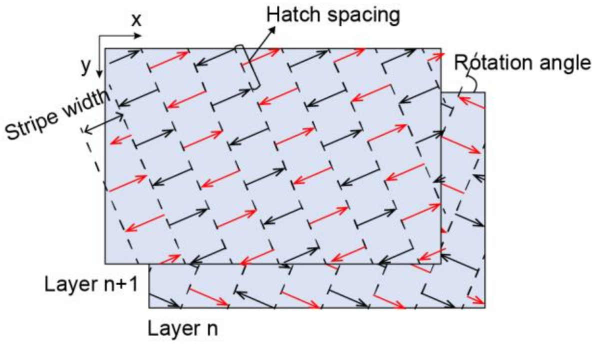

Figure S1. The stripe rotation scanning strategy was utilized for laser powder bed fusion manufacturing. The stripe width was 2 mms, the rotation angle was 67°, and the hatch spacing was 0.12 mm. The laser would change direction by 67 degrees in the next layer. Within a single layer, the laser scans in a specific sequence: first, in the region

indicated by the red arrow, followed by the region indicated by the black arrow. This pattern helps ensure uniform heating of the entire layer and reduces thermal stress.

2. Phase Transition Temperature Test

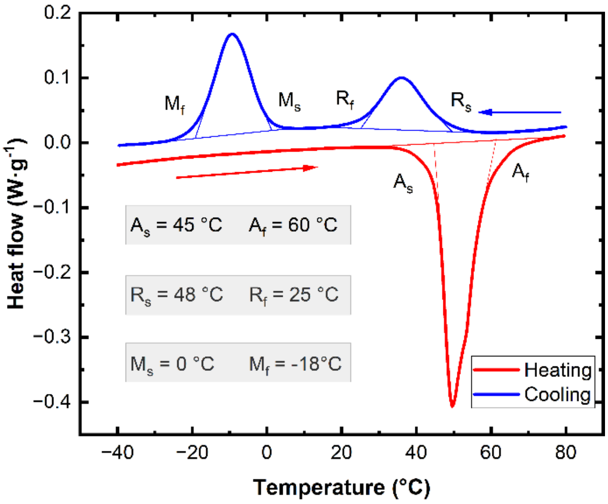

Figure S2. Differential scanning calorimetry (DSC) measurement results of the SMA sample fabricated by laser powder bed fusion. It is worth noting that during the cooling process, austenite first transforms into the rhombohedral phase (R-phase), and then further transforms into martensite. The formation of the intermediate phase, R-phase, is primarily influenced by the processing techniques. However, it does not destroy the shape memory effect and satisfies the requirements for use in this experiment.

3. Tensile testing of shape memory alloy samples

The dog-bone shaped samples (ASTM E8/E8M) with a gauge section of 6 mm in width, 2 mm in thickness, and 100 mm in length were printed to perform tensile tests in a universal testing machine (MTS 858) with a climate chamber. The experimental strain was determined based on the displacement of the testing machine actuator. The climate chamber used for temperature control precluded the use of a mechanical extensometer or optical system for strain measurement. The strain of the specimens was calculated as the effective strain, obtained by dividing the measured displacement by the initial length of the deforming components (excluding the clamping regions).

The specimens underwent tensile tests at -30 ℃ and 70 ℃ to characterize the

mechanical performance of NITI in its two states (martensite and austenite). The climate chamber temperature stabilized for 20 minutes after reaching the target temperature to ensure the phase transformation of the specimens was complete. The specimens were subjected to two-stage testing at low temperatures, involving strain-controlled loading (at a strain rate of 0.05% s-1) and stress-controlled unloading (at a stress rate of 1 MPa s-1), as illustrated in Figure S3a. During the loading process, the specimens went through three stages: the initial linear elastic stage (I) of twinned martensite, a plateau stage (II) associated with stress-induced martensite transformation, and the elastic deformation stage (III) of detwinned martensite. After unloading, the specimens exhibited residual strain. Subsequently, upon heating, the specimens underwent the transformation of detwinned martensite to austenite, resulting in the recovery of residual strain. This process represents the shape memory effect of NITI. When the specimens were in the austenite state, their elastic modulus increased. The specimens became stiffer but with reduced strength. The specimens fractured when the loading stress reached 640 MPa (Figure S3b).

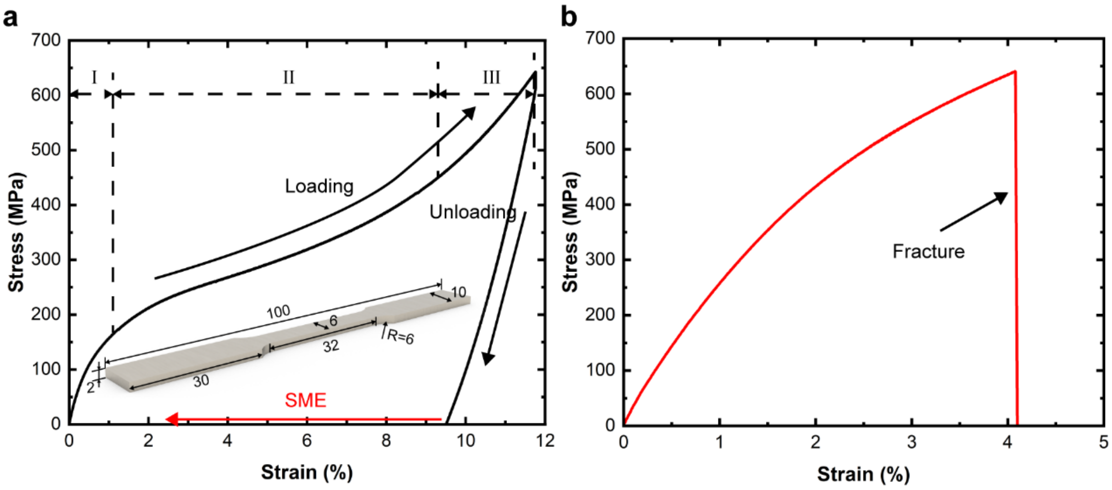

Figure S3. Mechanical property of LPBF-processed NiTi samples. (a) Tensile Stress-Strain Curve at Low Temperature (-30 °C, below M_f). (b) Tensile Stress-Strain Curve at High Temperature (70 °C, above A_f).

4. Influence of structural parameters on fabrication quality and performance

characteristics

The manuscript presents a prominent case study showcasing superior performance. Based on a conclusion derived from the model proposed in this paper, an initial high stiffness of the shape memory alloy netshell hinders its thermally induced self-recovery. Therefore, the initial structural design adopted a thinner thickness (T = 0.1 mm) and narrower width (W = 0.2 mm). However, due to the limitations of the SLM printing precision, structures that are too thin and narrow do not exhibit high fabrication quality and mechanical strength, as shown in Figure S4. After multiple printing tests, it was determined that good fabrication quality could be achieved with a thickness of 0.2 mm and a width of 0.4 mm.

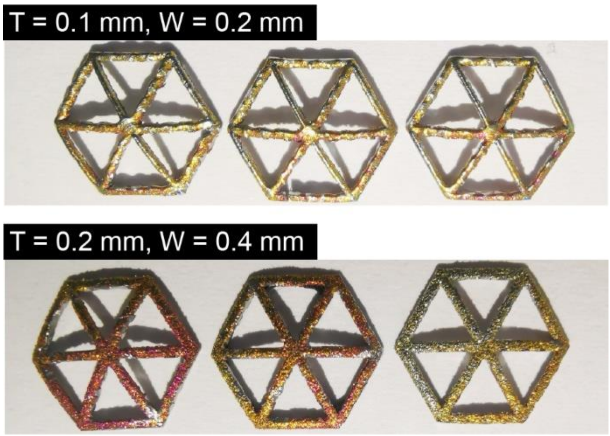

Figure S4. Influence of structural dimensions on manufacturing quality.

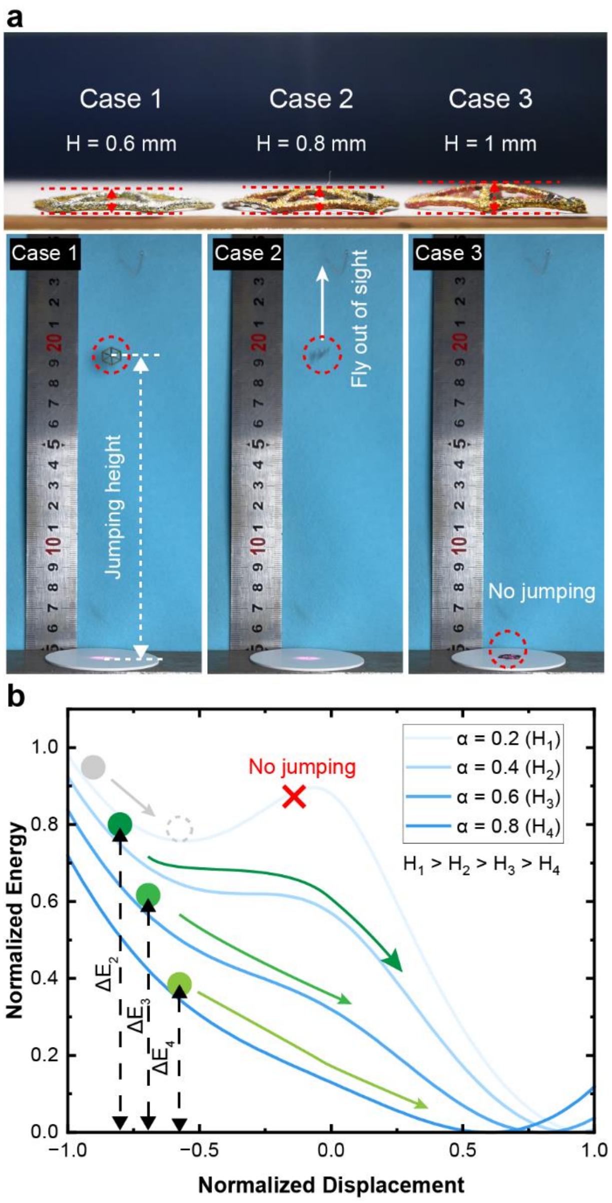

Figure S5. Effect of arch height on netshell jump performance. (a) The arch heights of the three samples were set to 0.6, 0.8, and 1 mm, respectively. Case 1 and Case 2 jumped under the optical drive, while Case 3 did not. Case 1 had a jump height of 140 mm. The maximum jump height for Case 2 exceeded the field of view. (b) Exploring the effect of arch height on the energy released during snapthrough using the analytical model proposed in this paper.

Subsequently, based on this structural design, we explored the influence of different arch heights (Figure S5a) on the netshell's snapping performance. Three cases had arch heights of 0.6, 0.8, and 1 mm, respectively. The model proposed in this paper can

deduce the impact of arch height on snapping performance (Figure S5b). Below a critical value, the energy released during the snap-through process increases with increasing height. However, when the arch height exceeds the critical value, the shape memory bistable system cannot spontaneously overcome the energy barrier to release energy. Experimental results indicate that Case 1 exhibits a lower jumping height (140 mm), Case 2 (the specimen presented in the manuscript) achieves a jumping height of 600 mm, and Case 3 does not exhibit jumping behavior, consistent with the conclusions drawn from the model. Nevertheless, the model presented in this paper can only provide qualitative conclusions. To obtain a more precise model description, we must address material and structural nonlinearity challenges. In future work, we will attempt to resolve this issue, providing more reliable theoretical guidance for structural optimization, such as determining the structural parameters for maximum release energy.

6. Influence of shape memory effect on netshell performance

We discussed the influence of arch height on the netshell above. Combining the proposed model allows us to elucidate how the shape memory effects affect netshell performance. The total system energy is divided into structural-related energy and shape memory-related energy, as shown in the following formula:

$$E_{norm}^{str} = \left(\sqrt{w_{norm}^2 + \alpha^2} - 1\right)^2 \tag{S1}$$

$$E_{norm}^{sm} = \frac{1}{2}\eta T_{norm}\left(\sqrt{1^2 - \alpha^2} - w_{norm}\right)^2 \tag{S2}$$

Here, E_{norm}^{str} represents the normalized structural-related energy, and E_{norm}^{sm} represents the normalized shape memory-related energy. The parameter α correlates with arch height. The smaller α is, the greater the arch height.

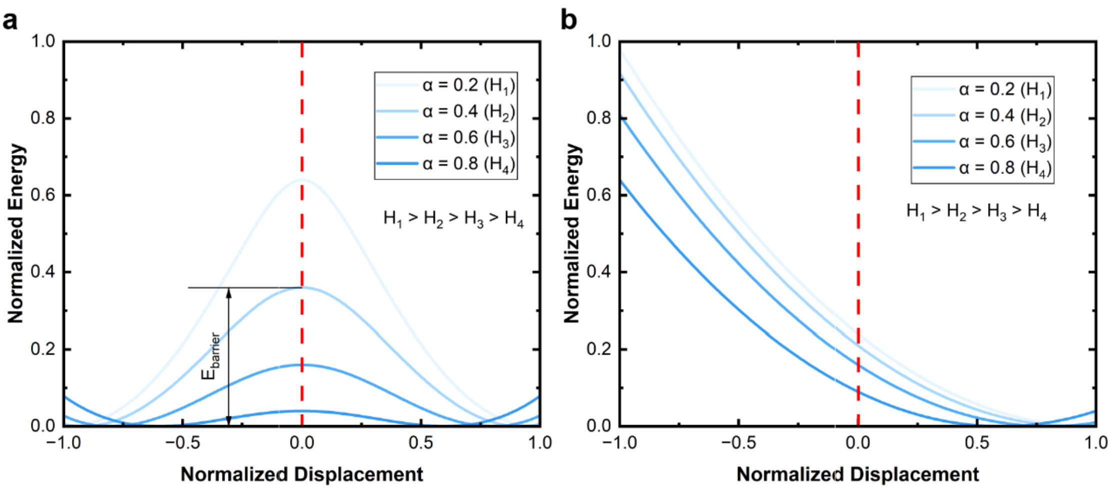

Figure S6. Effect of arch height on structural energy (a) and shape memory-related energy (b).

The structural-related energy exhibits a double-well configuration at different arch heights, which suppresses the system's thermal recoverability. The energy barrier for this configuration significantly increases with higher arch heights (Figure S6a). On the other hand, the shape memory-related energy maintains a single-well configuration at various arch heights, promoting the system's thermal recoverability. Therefore, the ultimate behavior of the entire system depends on the interplay between these two energies. When shape memory-related energy predominates, the system exhibits thermal recoverability. Vice versa, the system does not display thermal recovery. As illustrated in Figure S6b, shape-related energy strengthens with increasing arch height, but its sensitivity to arch height is not high. Consequently, when the arch height reaches a certain threshold, a dominant structural-related energy prevents the system from undergoing a snap-through transition. This qualitative analysis demonstrates the impact of shape memory effects on the behavior of the netshell.
